# Translational modelling of human microglia from cultures to chimeras

**DOI:** 10.1038/s12276-026-01866-1

**Published:** 2026-10-13

**Authors:** Jina Lee, Yongjin Yoo

**Affiliations:** 1 Department of Neuroscience, Korea University College of Medicine, Seoul, Republic of Korea; 2 BK21 Graduate Program, Department of Biomedical Sciences, Korea University College of Medicine, Seoul, Republic of Korea

**Keywords:** Microglia, Neurological models

## Abstract

Microglia play a crucial role in maintaining homeostasis of the central nervous system, whereas their dysfunction is implicated in the pathogenesis of various neurological diseases. Nevertheless, interspecific genetic differences pose challenges to translating findings from conventional rodent models. Human pluripotent stem cell-derived microglia offer a genetically relevant alternative, but these cells undergo profound transcriptomic changes and lose key homeostatic features when isolated from physiological brain cues. Accordingly, research has increasingly focused on advanced modelling platforms designed to restore the microenvironment required for in vivo microglial identity and functionality. This review examines the evolution of human microglial platforms, encompassing foundational generation protocols and multicellular integration. The discussion extends from two-dimensional co-cultures to three-dimensional brain organoids and includes human microglia chimeric models established via xenotransplantation to provide a physiological niche. In summary, the human pluripotent stem cell-based approach represents a valuable experimental pipeline for microglia research, facilitating the investigation of polygenic structures underlying human neurological diseases, from high-throughput screening to in vivo preclinical validation.

## Human Microglia Platforms Enhance Neurological Disease Research

Microglia, the resident immune cells of the central nervous system, play crucial roles in neural development and disease response. Recent studies highlight the limitations of rodent models due to genetic differences, prompting the development of human pluripotent stem cell-derived microglial models. These models, including planar co-cultures, three-dimensional organoids and chimeric brain xenografts, preserve human genetic features and offer controlled environments for research. The review discusses how these platforms advance neuroimmune research and therapeutic assessments. Results show that human pluripotent stem cell-derived microglia maintain homeostatic functions and respond to pathological stimuli, unlike their rodent counterparts. These findings are significant as they provide a more accurate representation of human microglial behaviour in disease contexts. The study advances our understanding of microglial biology and suggests future directions, such as integrating vascular networks and ageing protocols to better model human neurological diseases.

This summary was initially drafted using artificial intelligence, then revised and fact-checked by the author.

## Introduction

Microglia are the primary resident macrophages in the central nervous system (CNS), where they regulate neural development, sustain synaptic networks and respond to pathological stimuli. Genetic analyses have demonstrated that risk variants for neurodegenerative and neurodevelopmental disorders are enriched in microglial genes, underscoring the significance of microglia-mediated neuroinflammation in disease pathogenesis. Rodent models have historically contributed to foundational insights into microglial biology; however, interspecies genetic divergence restricts their translational relevance. Notably, numerous human-disease-associated risk variants lack functional orthologues in mice. Additionally, primary human microglia isolated from brain tissue undergo rapid transcriptomic alterations, losing homeostatic markers and exhibiting artefactual activation states under standard in vitro conditions. To overcome these challenges, human pluripotent stem cell (hPSC)-derived microglial models have been developed. Such platforms preserve human genetic features and enable the incorporation of microglia within controlled environments. This review summarizes the progression of hPSC-derived microglia models, spanning planar co-cultures, three-dimensional organoids and chimeric brain xenografts. Furthermore, we examine how these complementary platforms facilitate advances in neuroimmune research and preclinical therapeutic assessment.

## Species-specific divergence and microenvironmental dependency of microglia in health and disease

**Fig. 1:**
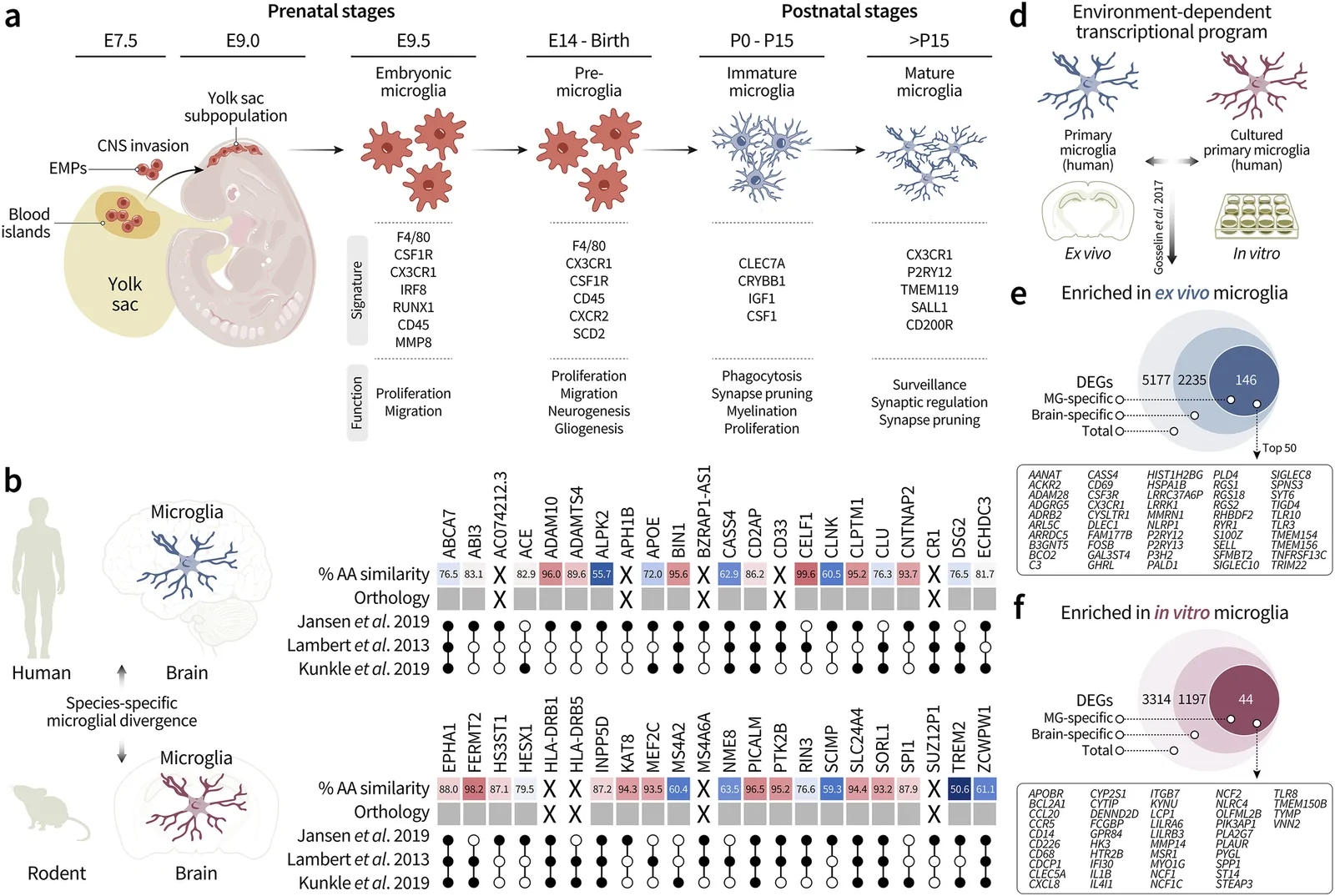
Ontogeny, interspecies divergence and environmental dependency of microglia. **a** Schematic depicting the developmental trajectory of microglia from yolk sac-derived erythromyeloid progenitors (EMPs). The timeline highlights key-stage-specific transcriptomic signatures and physiological functions across prenatal and postnatal maturation^75^. **b** Illustration highlighting the biological and genetic divergence between human and rodent microglia. **c** Comparative analysis of orthology and amino acid (AA) sequence homology for key microglial genes, emphasizing Alzheimer’s disease risk loci^76–78^. The colour gradient indicates the degree of sequence similarity from high (red) to low (blue), with crosses (X) denoting the absence of a direct murine orthologue. Data adapted from Mancuso et al.^13^. **d** The impact of microenvironmental cues on microglial identity. **e**, **f** Transcriptomic comparison between primary in vivo microglia and cultured ex vivo microglia, demonstrating the environment-dependent regulation of gene-expression programmes. List of differentially expressed genes (DEGs) obtained from Gosselin et al.^12^.

Microglia, the resident immune cells of the CNS, originate from yolk-sac-derived erythromyeloid progenitors during early embryogenesis and persist as a self-renewing population independent of definitive haematopoiesis^1,2^. Their maturation and maintenance rely on colony-stimulating factor 1 receptor (CSF1R) signalling and key transcription factors (TFs), notably PU.1 and IRF8 (ref.^3^) (Fig. 1a). In the healthy brain, microglia fulfil critical roles, including synaptic pruning via complement pathways, apoptotic-cell clearance and support for oligodendrocyte maturation through IGF1 secretion^4–9^. Signature genes — *P2RY12*, *TMEM119* and *CX3CR1* — are essential for preserving these homeostatic functions^10–12^ (Fig. 1a). Under pathological conditions, microglia transition from a homeostatic state to context-dependent reactive profiles. Early binary classifications such as “M1/M2” or “resting/activated” are now regarded as oversimplified. Advances in single-cell transcriptomics have elucidated a multidimensional spectrum comprising epigenetic, transcriptomic and metabolomic profiles, including homeostatic microglia (HM), disease-associated microglia (DAM), antigen-presenting (human leukocyte antigen [HLA]), cytokine-responsive (cytokine response modifier) and interferon-responsive states^13,14^. The DAM signature is characterized by downregulation of homeostatic markers, enhanced lipid processing and upregulation of risk gene expression in response to protein aggregates in Alzheimer’s disease (AD)^8,15,16^. Deficient microglial synaptic pruning during neurodevelopment correlates with disorders such as autism spectrum disorder (ASD) and schizophrenia^5,6,17^. Age-related changes render microglia primed, characterized by heightened inflammatory responses and reduced neuroprotective functions^18,19^.

Translating findings from animal models to humans is complicated by notable evolutionary divergence; human microglia demonstrate greater transcriptional heterogeneity and often express disease risk genes that are poorly conserved in murine models^13,20–23^. Approximately 41% of putative AD risk genes lack direct mouse orthologues, and even when present, sequence homology is frequently low^13,20,24,25^ (Fig. 1b, c). For instance, the *APOE* ε4 polymorphism, a major genetic risk factor for sporadic AD, is absent in rodents. Genes such as *CR1*, *CD33*, *TREM2* and members of the *MS4A* cluster either exhibit multiple orthologues or show less than 60% protein sequence identity^13,25^ (Fig. 1c). Such genetic discrepancies underscore the limitations inherent in rodent models for modelling human microglia-associated neurological diseases and advocate for the development of human microglial cellular models.

Establishing these platforms presents considerable challenges, as the cellular microenvironment influences microglial transcriptional plasticity. Isolated microglia rapidly adopt a pro-inflammatory phenotype ex vivo, causing in vitro monocultures to diverge markedly from in vivo microglia in transcriptomic profile^8,12,26–28^ (Fig. 1d). Transcriptomic analysis reveals that thousands of genes in human microglia alter their expression upon transition to in vitro conditions, with microglia-specific signatures particularly affected (Table 1, Fig. 1e, f). Ex vivo microglia retain homeostatic surveillance genes, such as *P2RY12*, *P2RY13*, *CX3CR1* and *SALL1*; cultured microglia instead upregulate broad inflammatory and phagocytic programmes, including pro-inflammatory cytokines (*IL1B*, *CXCL8*, *CCL20*), chemokine receptors (*CCR5*), antigen presentation factors (*CD68*, *IFI30*) and NADPH oxidase components (*NCF1*, *NCF2*; Fig. 1e, f).

**Table 1:** Environment-dependent transcriptional changes in human microglia.

Gene symbol	Function	Refs.
**Downregulated in vitro compared with in vivo**		
*P2RY12*	ATP sensing, process motility	^13,14^
*TMEM119*	Microglial identity marker	^13^
*CX3CR1*	Neuron-microglia communication	^13,14^
*P2RY13*	ADP receptor	^14^
*GPR34*	GPCR signalling	^12^
*SALL1*	Transcription factor	^13^
*MERTK*	Phagocytosis of apoptotic cells	^33^
*PROS1*	TAM receptor ligand	^33^
*HEXB*	Lysosomal enzyme	^12^
**Upregulated in vitro compared with in vivo**		
*IL1B*	Pro-inflammatory cytokine	^14^
*TNF*	Pro-inflammatory cytokine	^12^
*CXCL10*	Chemokine, T cell recruitment	^13^
*CCL3*	Chemokine (CRM marker)	^14^
*CCL4*	Chemokine (CRM marker)	^14^
*MMP9*	Matrix metalloproteinase	^12^
*CD68*	Lysosomal marker	^33^
*APOE*	Lipid metabolism (context-dependent)	^13,14^

*CRM* cytokine response microglia.

This bidirectional shift demonstrates how the absence of CNS environmental cues propels microglia towards a chronic activation state distinct from in vivo phenotypes^12,28^. Co-culture with neurons, astrocytes and oligodendrocytes partially rescues homeostatic gene expression, facilitated by paracrine signals, including transforming growth factor (TGF) β, interleukin (IL)-34, colony-stimulating factor 1 (CSF1) and cholesterol metabolites. Xenotransplantation of hPSC-derived microglia into the mouse brain further drives maturation towards a transcriptomic profile closely aligned with that of primary human microglia, with minimal differential gene expression^13,29–31^. This phenotypic convergence underscores the role of three-dimensional tissue architecture and cellular diversity in maintaining microglial identity.

Current research efforts thus focus on developing hPSC-derived microglial models that preserve human-specific genetic backgrounds and recapitulate in vivo-like signatures. These platforms enable the detailed investigation of microglia-mediated neuroinflammation and aberrant phagocytosis, as well as the interplay between polygenic risk factors such as triggering receptor expressed on myeloid cells 2 (TREM2), and the local brain microenvironment — factors often obscured in traditional rodent models. Additionally, they support high-throughput screening of immunomodulatory therapeutics targeting human-specific microglial receptors.

## Approaches to generating and characterizing human pluripotent stem cell-derived microglia

**Fig. 2:**
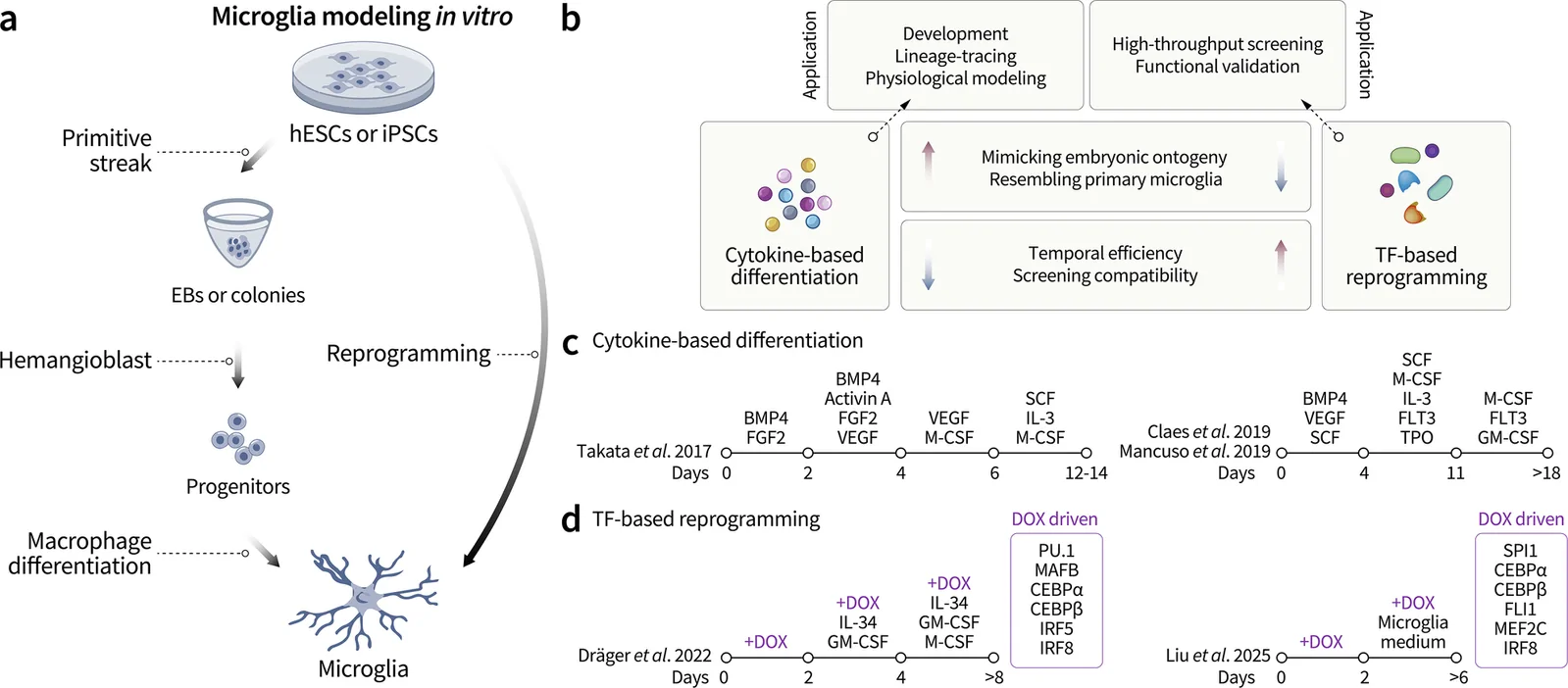
Generation of human pluripotent stem-cell-derived microglia via directed differentiation or direct reprogramming approaches. **a** Schematic overview depicting the two primary approaches for generating microglia from human pluripotent stem cells. These approaches include either directed differentiation utilizing defined cytokine supplementation or direct reprogramming driven by the forced expression of transcription factors (TF). **b** Methodological trade-offs and experimental application fields of human microglial models. Cytokine-based differentiation mimics embryonic ontogeny to closely resemble primary microglia, whereas TF-based reprogramming provides temporal efficiency and compatibility with high-throughput genetic screening. **c**, **d** Timelines and specific regimens of representative recent protocols. **c** Cytokine-based stepwise differentiation approaches sequentially expose cells to stage-specific morphogens and survival factors to derive microglia. **d** TF-mediated direct reprogramming approaches rely on the doxycycline (DOX)-driven inducible expression of key myeloid lineage-determining factors to generate microglia-like cells within days. EBs, embryonic bodies; hESCs, human embryonic stem cells; iPSCs, induced pluripotent stem cells.

The generation of functional microglia from hPSCs is accomplished through two complementary methodologies. The cytokine-based differentiation recapitulates yolk-sac haematopoiesis (Table 2, Fig. 2a), whereas TF-mediated direct lineage conversion drives rapid fate determination (Table 3, Fig. 2a). Each method offers unique advantages in scalability, purity, culture duration and developmental fidelity.

**Table 2:** Current approaches to generating human pluripotent stem-cell (hPSC)-derived microglia via directed differentiation.

Highlight	Generated cell type	Cellular origin	Methodologies	Characterization	Refs.
**Embryonic bodies-based differentiation**					
Characterization of iPSC-derived microglia and establishment of pMGLs using disease-specific cell lines	pMGL (PSC-derived microglia-like cells)	hESC, iPSC	Induction relies on yolk-sac-like embryoid body formation in neuroglia differentiation medium containing CSF1 and IL-34 Subsequent maturation uses low CSF1 and high IL-34 concentrations	IBA1, CD11b, PU.1, CD45	^33^
Differentiation of macrophage into tissue-resident macrophage and mimicking the disease phenotype	iMac (induced macrophage) iMicro (induced microglia)	iPSC	Stepwise application of mesodermal and haematopoietic cytokines followed by CSF1 yields primitive macrophages Terminal microglial differentiation is achieved by isolating CD45^+^CD11b^+^CD163^+^CD14^+^CX3CR1^+^ progenitors and co-culturing with neurons	IBA1	^36^
iPSC-derived neurons and microglia co-culture has specific cytokine profiles	pMacpre (PSC-derived macrophage/microglia precursors) pMac: PSC-derived macrophages pMGL: PSC-derived microglia-like cells	iPSC	Macrophage precursors are generated through sequential exposure to BMP4, VEGF, SCF, M-CSF and IL-3 Subsequent microglial maturation is induced using GM-CSF and IL-34 in monoculture or neuronal co-culture	IBA1, CD11b, CD14, CD45, MERTK	^70^
**Two-dimensional colonies-based differentiation**					
iPSC differentiation into microglia-like cells by co-culture with astrocytes		iPSC	Haematopoietic progenitors are generated either through OP9 stromal co-culture or feeder-free induction using early morphogens Terminal microglial maturation is achieved by co-culturing these progenitors with astrocytes supplemented with GM-CSF, M-CSF and IL-3 followed by marker-based isolation	CD11b, HLA-DR, IBA1, CD45, TREM-2, CX3CR1	^32^
iPSC-MG are similar with human primary microglia in gene expression and cytokine release	iPSC-MG	hPSC	Stepwise application of mesodermal and haematopoietic cytokines yields myeloid progenitors Terminal microglial differentiation is achieved by isolating CD14^+^ or CD14^+^CX3CR1^+^ cells and culturing them with GM-CSF and IL-34	IBA1, CD11c, TMEM119, P2RY12, C1QA, GAS6, GPR34, MERTK, PROS1	^35^
AD-related microglia study via iMGL transplantation in mice and human brain organoids	iHPC (iPSC-derived haematopoietic progenitors) iMGL (iPSC-derived microglial-like cells)	iPSC	Haematopoietic progenitors are generated through sequential exposure to mesodermal and haematopoietic cytokines Subsequent microglial differentiation is achieved by isolating CD43^+^ cells and culturing with M-CSF, IL-34, and TGFβ followed by terminal maturation with CD200 and CX3CL1	PU.1, TREM2, P2RY12	^34^
PSC-derived microglia with TREM2^R47H^ mutation have phagocytic defects	tMGs (transdifferentiated microglia-like cells)	hESC	Monocytes are generated through sequential exposure to mesodermal and haematopoietic cytokines Monocytes are subsequently transdifferentiated into microglia-like cells using IL-34 and M-CSF	P2RY12, PROS1, TGFBR1, OLFML3, C1Q, CSF1R, TMEM119, APOE, TREM2	^38^
Human PSC-derived microglia transplantation into mouse brain and comparison with mouse microglial response of oligomeric amyloid-β	In vitro MG In vivo HM	hESC	CD14^+^ monocytes are generated and isolated from pluripotent stem cells These monocytes are differentiated into microglia-like cells using a specific combination of IL-34, M-CSF, CX3CL1 and TGFβ	CX3CR1, P2RY12	^13^

*AD*, Alzheimer’s disease; *hESC*, human embryonic stem cell; *HM*, homeostatic microglia; *iPSC*, induced pluripotent stem cells; *PSC*, pluripotent stem cells.

**Table 3:** Transcription factor-mediated reprogramming of human pluripotent stem cells into microglia-like cells.

Generated cell type stated	Transcription factors	Methodologies	Characterization	Refs.
iMG (iPSC-derived microglia-like cells)	SPI1, CEBPA	Microglial conversion is driven by doxycycline-induced expression of SPI1 and CEBPA alongside sequential exposure to early morphogens and haematopoietic cytokines Transduced cells are enriched via puromycin selection and terminally matured using IL-34, M-CSF, GM-CSF, and TGFβ1	IBA1, CD11b, TREM2, CX3CR1, TMEM119, C1QA, GPR34, P2RY12, PU.1	^40^
hiHPCs (haematopoietic progenitor cells from hiPSCs) hiMGLs (human iPSC-derived microglia)	SPI1	Haematopoietic progenitors are generated through a doxycycline-inducible PiggyBac system combined with sequential exposure to early morphogens and haematopoietic cytokines Subsequent microglial differentiation and maturation are achieved using IL-34, TGFβ, M-CSF, CD200, and CX3CL1	CX3CR1, IBA1, PU.1, TMEM119, P2RY12, TREM2, DAP12	^39^
hiMGLs (iPSC-derived microglia)	SPI1, CEBPB	Microglial conversion is driven by a dual genomic safe harbour doxycycline-inducible system alongside sequential exposure to early morphogens and hematopoietic cytokines Floating precursors are subsequently collected and terminally matured using M-CSF, IL-34, and TGFβ1	CD11b, CD45, CX3CR1, CD11c, CD14, CD16, ENTPD, P2RY12, TREM2, TMEM119, IBA1, GAS6, MERTK, C1QA, PROS1, GPR34, SALL1	^7^
iTF-Microglia (induced-transcription factor microglia-like cells)	MAFB, CEBPA, IRF8, PU.1, CEBPβ, IRF5	Microglial conversion is driven by doxycycline-induced expression of PU.1, CEBPα, CEBPβ, IRF5, IRF8 and MAFB integrated into genomic safe harbours Terminal maturation is subsequently achieved using IL-34, GM-CSF, M-CSF and TGFβ1	GPR34, IBA1	^41^
TF-iMGLs (MG6.4-SCF-MCI TF-induced MicroGlial-Like cells)	SPI1, CEBPA, FLI1, MEF2C, CEBPB and IRF8	Iterative screening identified a six-TFs combination comprising SPI1, FLI1, CEBPA, MEF2C, CEBPB and IRF8 Microglial conversion is driven solely by doxycycline-induced expression of these TFs	CD11b, P2RY12, CX3CR1	^42^

*iPSC*, induced pluripotent stem cell; *TF*, transcription factor.

Cytokine-driven protocols are designed to closely mimic embryonic ontogeny (Fig. 2b, c). Early strategies reconstructed the emergence of erythromyeloid progenitors from the yolk sac, either by facilitating the spontaneous release of primitive myeloid precursors from embryoid bodies or by employing stepwise two-dimensional adherent cultures with key morphogens (bone morphogenetic protein 4 [BMP4], vascular endothelial growth factor [VEGF], stem-cell factor [SCF])^32,33^. However, these early ontogeny-guided approaches often necessitate prolonged culture times, astrocyte co-cultures or serum-containing media that can artefactually activate microglia. Hence, more recent techniques have adopted chemically defined, serum-free conditions^26,33,34^. The inclusion of hypoxic incubation more accurately reflects the physiological oxygen environment of the developing embryonic niche^35,36^. Refinements, including the temporal delivery of terminal survival factors — TGFβ, macrophage colony-stimulating factor (M-CSF) and IL-34 — ultimately obviate the requirement for manual cell sorting and feeder layers^13,34,37,38^. These refined protocols improve reproducibility and facilitate the scalable production of pure microglia-like populations.

In contrast, direct lineage conversion circumvents intermediate developmental stages, promoting accelerated fate specification and mitigating the extended timelines inherent to cytokine-based methodologies (Table 3, Fig. 2b, d). Early protocols relied on inducible overexpression of single regulators such as PU.1, which accelerates haematopoietic commitment yet still requires weeks for terminal maturation^39^. More recent methods utilize multi-factor cocktails — including PU.1, CEBPα, CEBPβ, interferon regulatory factor (IRF)5, IRF8 and either MAFB or FLI1 — to activate a microglial transcriptional network within days^7,40–42^ (Table 3, Fig. 2d). Later iterations involve targeted integration into genomic safe harbours (e.g. *AAVS1*, *CLYBL* loci) to avoid random viral integration, thereby ensuring stable and uniform TF cassette expression without disrupting endogenous gene function. The rapid induction kinetics and uniformity of this combinatorial strategy make it particularly well suited to pooled CRISPR screening applications to systematically elucidate regulators of microglial disease state^41,43^.

Advancements in understanding stage-specific transcriptional networks now support tailored microglial reprogramming. High-resolution mapping reveals that distinct regulatory networks mediate the progression from embryonic to adult microglial phenotypes^44^. Persistent core regulators such as PU.1 act alongside fetal-state drivers (MITF family, MAFB), whereas late-stage networks (IRF, KLF and STAT families) facilitate the acquisition of mature postnatal phenotypes^44^. This evolving regulatory framework provides a conceptual basis for refining TF cocktails, in which overexpression of specific IRF, KLF and STAT proteins may induce a mature postnatal signature, whereas excessive reliance on early-stage drivers (MAFB, MITF) can accelerate induction but impede full-maturation phenotypes^44^. Leveraging these stage-specific regulatory networks represents a promising approach to overcoming the fetal-like limitations of current hPSC-derived microglial cultures.

These two methodological frameworks present distinct trade-offs (Fig. 2b). Cytokine-driven strategies, which recapitulate embryonic ontogeny, yield cells with homeostatic profiles closely aligned with those of primary human microglia, serving as robust models for developmental studies and investigations into neuroimmune interactions. In comparison, TF-mediated reprogramming is efficient, generating cells with fetal-like microglial transcriptional profiles and enabling pooled CRISPR screening to systematically study microglial disease regulators and validate polygenic risk variants. Nevertheless, both approaches yield functional human microglia applicable to in vitro modelling of key activities, including amyloid-β phagocytosis, purinergic calcium signalling and disease-specific inflammatory responses.

## Advancing spatial complexity of in vitro microglial models: from monolayer cultures to brain organoids

**Fig. 3:**
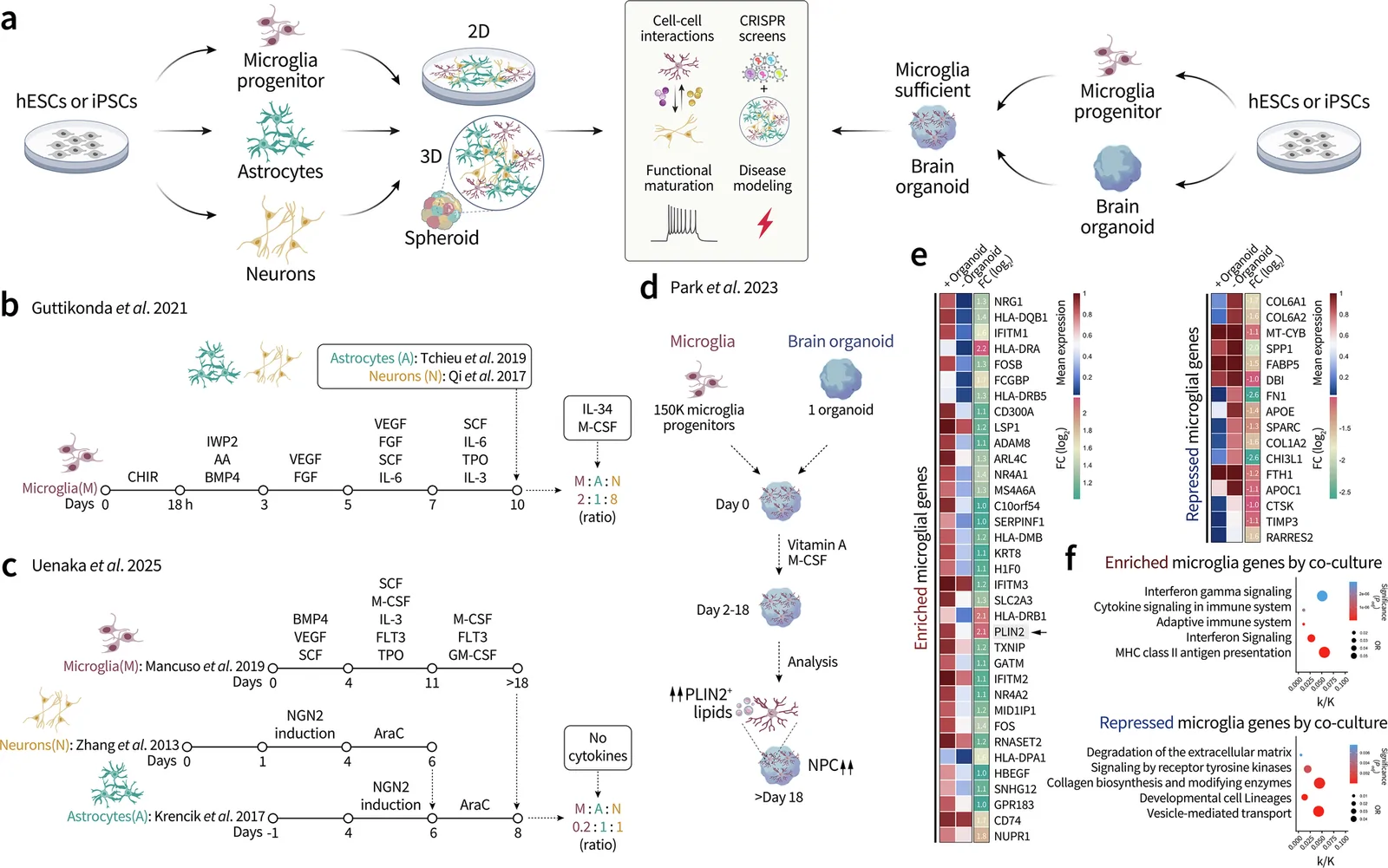
Human microglia co-culture systems and their applications in functional studies and disease modelling. **a** Overview of microglial integration platforms and experimental applications. Human pluripotent stem cell-derived microglial progenitors are combined with neurons and astrocytes to form two-dimensional co-cultures, three-dimensional spheroids, or integrated into brain organoids. These platforms support a range of applications, including cell–cell interaction studies, CRISPR screens, functional maturation analysis and disease modelling. **b** Schematic overview of a cytokine-dependent tri-culture system adapted from Guttikonda et al.^46^. The timeline details the differentiation of microglia, astrocytes and neurons using defined cytokines and growth factors prior to co-culture. **c** Schematic overview of a cytokine-independent tri-culture system adapted from Uenaka et al.^47,79,80^. This approach achieves cytokine independence by utilizing endogenous factors, such as M-CSF, secreted by mature astrocytes to support microglial survival. **d** Experimental scheme detailing the integration of hPSC-derived microglia into cortical organoids adapted from Park et al.^50^, highlighting microglial PLIN2^+^ lipid droplet accumulation and the regulation of neural progenitor cells (NPCs). **e** Heatmap illustrating the enrichment or repression of microglial genes upon organoid co-culture (data adapted and replotted from Park et al.^50^). **f** Pathway (Reactome) enrichment analysis of microglial gene sets significantly modulated by integration into the organoid environment. hESC, human embryonic stem cell; iPSC, induced pluripotent stem cell.

Microglial functionality is intrinsically linked to ongoing reciprocal signalling with neurons, astrocytes and the extracellular matrix. When cultured in isolation, microglia rapidly lose core homeostatic markers and instead acquire pro-inflammatory profiles^26^, underscoring the influence of environment-dependent transcriptomic changes. This phenomenon highlights the necessity of co-culture methodologies to better recapitulate the in vivo milieu. Accordingly, hPSC-based models have evolved from planar monolayer cultures to three-dimensional organoid systems (Tables 4, 5, Fig. 3a), each offering distinct advantages regarding microenvironmental cues and experimental parameters.

**Table 4:** Tri-culture systems of human pluripotent stem cell-derived neurons, astrocytes and microglia.

Cell types	Neuron/astrocyte generation	Methodologies for microglia engagement	Characterization of microglia	Refs.
iPSC-NPC, microglia	Neural progenitor cells carrying Alzheimer’s disease mutations are differentiated into neurons and astrocytes within a three-dimensional microfluidic platform	Immortalized SV40 microglia are subsequently introduced to establish a triculture system and assess microglial engagement	CD68, CD11b	^45^
-	Neural progenitor cells are induced using LDN193189, SB431542 and XAV939 followed by neuronal maturation with neurotrophic factors; astrocytes are independently generated from neural stem cells through NFIA induction and subsequent sorting of CD44^+^ progenitors	CD45^+^ macrophage precursors are generated from KDR^+^CD235A^+^ haemangioblasts through sequential exposure to early morphogens and hematopoietic cytokines Terminal microglial maturation is achieved using IL-34 and M-CSF in either monoculture or cortical neuron co-culture	IBA1, PU.1, CD45, CX3CR1	^46^
iNs (induced neuronal cells), astrocytes, iMG (induced microglia)	Neurons are rapidly generated via Ngn2 induction whereas cortical-like astrocytes are independently differentiated	Microglial progenitors are generated through sequential exposure to early morphogens and haematopoietic cytokines followed by isolation of CD14^+^ or CD14^+^CX3CR1^+^ cells A tri-lineage co-culture is subsequently established by sequentially plating mature astrocytes, Ngn2-induced neurons and the isolated microglia	IBA1, CD14, CX3CR1	^47^
iNs (induced neuronal cells), iA (induced astrocyte), iMG (induced microglia)	Neurons and astrocytes are rapidly generated via NGN2 and SOX9/NFIB induction respectively	Microglia are generated from haematopoietic precursors using IL-34, M-CSF and TGFβ1 followed by terminal maturation with CX3CL1 and CD200 These mature microglia are subsequently integrated into a triculture system alongside induced neurons and astrocytes	IBA1, P2RY12	^48^

**Table 5:** Current approaches to integrating microglia into brain organoids.

Generated cell type and system stated	Organoid generation	Brief methodologies for microglia engagement	Characterization	Refs.
iMG (induced microglia), three-dimensional neuroimmune cerebral organoid model	^71^	Microglia are derived using established differentiation protocols or isolated from primary tissue A three-dimensional neuroimmune model is subsequently established by engrafting the primary human microglia into cerebral organoids	IBA1	^51^
hCOs (human cortical organoids), mhCOs (microglia-containing hCOs)	^72^	Microglia-containing cerebral organoids are generated by mixing wild-type hPSCs with a minor subpopulation of PU.1-transduced cells Subsequent doxycycline treatment induces PU.1 expression and drives endogenous microglial development within the three-dimensional architecture	IBA1, CSF1R, TMEM119, P2RY12	^53^
iMac, iMicro, NPC	^73^	Microglia-sufficient brain organoids are generated by co-culturing induced primitive macrophages with cerebral organoids The integrated microglial population is subsequently maintained using CSF-1 supplementation	P2RY12, TMEM119, CX3CR1, SALL1, IRF8, FOS, EGR3, CEBPD, JUN, MAFF	^50^
Forebrain organoids, EMPs (erythromyeloid progenitors), hMGs (human microglia), iHBO (immunocompetent human brain organoid)	^74^	Isolated CD43^+^ erythromyeloid progenitors are co-cultured with cortical organoids using M-CSF, IL-34, and TGFβ1 The resulting immunocompetent forebrain organoids are subsequently xenotransplanted into a mouse brain	IBA1, PU.1, TMEM119, P2RY12	^52^
hCO-Mg (human cortical organoids with microglia), hStrO-Mg (human striatal organoids with microglia), hMO-Mg (human midbrain organoids with microglia) hCO-hStrO-microglia assembloid, hMO-hStrO-microglia assembloid	Region-specific cortical organoids are generated through dual-SMAD and Wnt inhibition followed by maturation with neurotrophic factors; striatal and midbrain organoids are independently derived following established differentiation methods	Microglia are differentiated from haematopoietic progenitors using IL-34, TGFβ1 and M-CSF and incorporated into region-specific cortical, striatal or midbrain organoids These structures are subsequently fused to establish multi-regional microglial-integrated assembloids	IBA1, CSF1R, CD68	^54^

Initial efforts to model neuro-immune dynamics relied on two-dimensional co-cultures and tri-cultures comprising hPSC-derived neurons, astrocytes and microglia (Table 4). These planar systems facilitate refined genetic manipulation, high-throughput screening and high-resolution imaging of subcellular processes. For example, microfluidic devices provide valuable insights into microglial migration in response to pathological neural tissue characterized by the aggregation of amyloid β and phosphorylated tau^45^. Defined tri-culture systems have revealed fundamental mechanisms of intercellular communication^46^ (Fig. 3b). Notably, co-maturation models demonstrate that astrocytes are essential for microglial survival by secreting factors such as CSF1 (ref.^47^) (Fig. 3c). Moreover, acute exposure to familial AD neurons in tri-culture systems suppresses microglial disease-associated signatures typically induced by astrocytes^48^. Despite yielding valuable functional insights, monolayer systems enforce artificial planar interactions and fail to mimic the brain’s viscoelastic matrix. Such constraints prevent physiological maturation and limit architectural complexity^45,49^.

To address these limitations, three-dimensional brain organoid models have been adopted that embed microglia within a neuroectodermal matrix containing diverse neural progenitors and glia^50–53^ (Table 5, Fig. 3a). Integration strategies include the exogenous addition of primitive macrophage progenitors and the endogenous co-differentiation of microglia via targeted overexpression of myeloid-specific TFs (e.g. PU.1) in selected pluripotent stem-cell subsets^53^. The efficiency with which exogenous myeloid cells colonize host tissues is contingent on developmental timing, with optimal integration at the onset of neurogenesis^52^.

Single-cell transcriptomic analyses demonstrate that the three-dimensional organoid microenvironment preserves microglial homeostatic states, yielding cells that more closely resemble their primary in vivo counterparts than standard two-dimensional cultures^51,52^. Importantly, integrated microglia modulate the developmental trajectories of adjacent neural-cell populations by attenuating IRF gene expression and reducing cellular stress markers, including double-stranded DNA breaks in radial glia^51^.

Microglia-sufficient organoids exhibit a lipid-mediated crosstalk mechanism, as established by experimentally integrating hPSC-derived microglial progenitors into cortical organoids^50^ (Fig. 3d). Upon organoid integration, microglia accumulate PLIN2^+^ lipid droplets and export cholesterol, which is taken up by neural progenitor cells, triggering a metabolic reprogramming that limits excessive proliferation while promoting axonogenesis and neuronal maturation^50^. Comparative transcriptomic analysis between organoids with and without integrated microglia emphasizes broad reprogramming effects (Fig. 3e), including upregulation of immune-related pathways (IFN signalling, cytokine signalling, MHC class II presentation) and downregulation of extracellular matrix remodelling, collagen biosynthesis and receptor tyrosine kinase signalling (Fig. 3f). Collectively, these observations suggest that integrated microglia confer simultaneous immunocompetence and developmental modulation within neural organoids^50^.

Beyond neurodevelopmental studies, microglia-integrated organoid models are instrumental for neurodegenerative disease research. For instance, microglia protect neural circuits from amyloid β toxicity by mitigating ferroptosis and suppressing disease-associated transcriptomic abnormalities^53^. These complex systems also enable pooled CRISPR interference screening to systematically interrogate genetic risk factors for neurodegeneration in situ^53^.

Multi-regional brain assembloids extend this paradigm, enabling investigation of microglial spatial heterogeneity and circuit-level interactions^54^. Integrated microglia adopt niche-specific transcriptomic signatures characterized by GABA_B_ receptor enrichment within striatal regions and support long-range axonal wiring in midbrain-striatal assembloids. Such models facilitate interrogation of neuropsychiatric mechanisms; e.g. introduction of autism-associated *SCN2A* mutations induces neuronal hyperexcitability, triggering GABA_B_ receptor-dependent microglial pruning of inhibitory synapses^54^. Reversing this pathology via targeted GABA_B_ receptor inhibition illustrates how microglia shape neurodevelopmental circuits and how such pathology can be therapeutically targeted.

Despite these advances, considerable challenges remain. Monolayers lack cytoarchitectural complexity, impede physiological maturation and fail to prevent transcriptomic drift. In contrast, microglia–organoid integration systems are hindered by inter-batch variability, inconsistent microglial distribution and compromised survival during extended culture, often leading to loss of mature homeostatic signatures^52^. Moreover, both platforms predominantly generate microglia, retaining fetal-like or early postnatal transcriptomic signatures, complicating the accurate modelling of ageing-related disorders.

Future research must address these bottlenecks by engineering three-dimensional scaffolds that mimic brain architecture to sustain homeostatic microglial morphologies. Additionally, developing automated microfluidic systems will enable tracking of microglial chemotaxis. Furthermore, incorporating vascular networks prevents hypoxia-induced artefactual microglial activation within the necrotic cores of organoids and restores the perivascular niche, thereby emulating systemic immune crosstalk. Finally, applying accelerated ageing protocols will mature these fetal-like cells into aged microglial phenotypes essential for studying ageing-associated neurological diseases.

## Human microglial xenotransplantation: a translational platform for modeling neurological diseases and evaluating therapeutic interventions

**Fig. 4:**
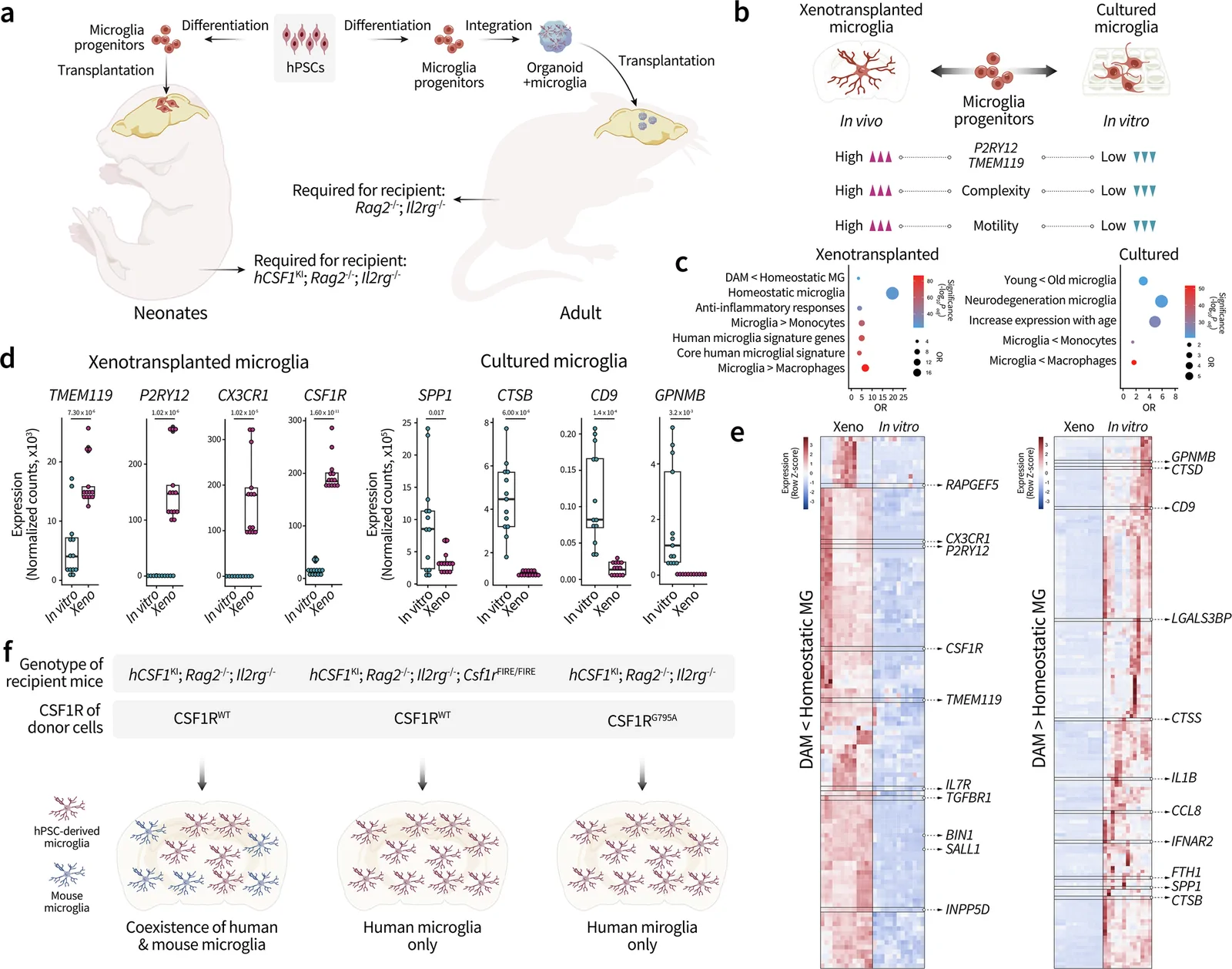
Human pluripotent stem-cell-derived human microglia in the mouse brain closely resemble primary human microglia. **a** Schematic overview of xenotransplantation strategies. Human pluripotent stem-cell (hPSC)-derived microglial progenitors can be directly injected into the brains of neonatal mice or integrated into brain organoids prior to transplantation into adult mouse brain. Both paradigms utilize immunodeficient (*Rag2*^−/−^; *Il2rg*^−/−^) mouse models with or without expressing humanized CSF1 (hCSF1-KI) to prevent immune rejection and provide species-specific trophic support. **b** Morphological and phenotypic comparison between in vitro cultured and xenotransplanted hPSC-derived microglia. Unlike standard cultures, which exhibit reduced morphological complexity and diminished homeostatic marker expression, xenografted microglia acquire a ramified morphology, characteristic surveillance motility and robust expression of core markers. **c** Gene-set enrichment analysis comparing the transcriptomic profiles of cultured and xenotransplanted microglia. Engrafted cells are enriched for homeostatic, anti-inflammatory and core human microglial signatures, whereas in vitro cultured cells predominantly express disease-associated microglia (DAM), neurodegeneration, and ageing-associated transcriptional programmes. **d** Quantification of key microglial gene expression. Xenotransplantation rescues the expression of canonical homeostatic genes (e.g. *TMEM119*, *P2RY12*, *CX3CR1*, *CSF1R*), while downregulating activation and disease-associated markers (e.g. *SPP1*, *CTSB*, *CD9*, *GPNMB*) that are elevated under in vitro conditions. **e** Transcriptomic heatmap illustrating the distinct gene expression profiles of cultured versus xenotransplanted microglia, highlighting the shift from an artefactual culture-induced state to a physiological in vivo homeostatic signature. **f** Methodological advances for controlling microglial chimerism and overcoming spatial limitations. Standard transplantation of CSF1R wild type hPSC-microglia into regular hosts yields a mosaic coexistence of human and mouse cells. Utilizing host mice genetically lacking endogenous microglia (e.g. *Csf1r*^ΔFIRE/ΔFIRE^) or engineering hPSC-microglia with a CSF1R-inhibitor resistance mutation (*CSF1R*^G795A^) coupled with pharmacological depletion enables near-complete replacement of the murine microglial compartment across the brain^59^.

Although in vitro systems provide valuable insights, they often fail to reconstruct the complex CNS niche. Xenotransplantation of human microglia into mouse brains creates an in vivo three-dimensional tissue environment that supports cellular development (Fig. 4). This chimeric platform preserves experimental tractability by enabling genetic manipulation of human progenitors prior to engraftment, followed by evaluation within controlled in vivo disease models. This strategy reveals species-specific microglial responses and enables the investigation of human genetic risk variants that lack functional mouse orthologues^13,25^.

Successful engraftment of hPSC-derived microglia into the rodent CNS requires overcoming immunological rejection and species-specific growth factor incompatibility. Deleting *Rag2* and *Il2rg* abolishes adaptive immunity and natural killer-cell activity, preventing rejection^55^. Given that murine CSF1 binds poorly to human CSF1R, “humanization” of CSF1 (hCSF1) is required for the survival and development of the transplanted cells^56^ (Fig. 4a). Hence, the Rag2^−/−^ Il2rg^−/−^ *hCSF1*^KI^ (hCSF1-KI) mouse model, which expresses human CSF1 under the mouse promoter, provides controlled trophic support and serves as a robust platform for studying human myeloid cells^13,27,30,31,56^. Although early models^29,57^ such as MITRG mice expressed several human cytokines (hCSF1, IL-3, GM-CSF, SIRPα and TPO), more recent data have demonstrated that hCSF1 alone is sufficient to sustain robust microglial engraftment^29^. This streamlined model minimizes potential confounding effects from the overexpression of additional cytokines^13,14,29,30,58^.

Standard xenotransplantation entails the intracerebroventricular (ICV) injection of hPSC-derived myeloid progenitors into hCSF1-KI mouse neonates^13,31^ (Fig. 4a). Pre-treating these neonates with a CSF1R inhibitor temporarily depletes their own microglia and reduces competition for survival signals, thereby creating a permissive engraftment niche^13,30,31^. Human microglia constitute ~30% of all microglia 3 weeks post-transplantation and can expand to 80% chimerism after 3–6 months, as they gradually outcompete residual murine microglia^13,30,31^. The brain thus becomes a mosaic of human and mouse microglia, allowing direct within-animal comparisons of their responses to disease^14^.

Within the mouse CNS, transplanted human microglial progenitors mature and adopt transcriptomic profiles similar to those of primary human microglia^13,30,31^. Gene-set enrichment analysis demonstrates that these engrafted cells acquire homeostatic, anti-inflammatory and core human microglial signatures — unlike cultured cells, which show neurodegenerative and ageing-associated gene patterns and lose core identity (Fig. 4b, c). Canonical homeostatic genes such as *TMEM119*, *P2RY12*, *CX3CR1* and *CSF1R* are restored to physiological levels in xenografted microglia, whereas activation and disease-associated markers — including *SPP1*, *CTSB*, *CD9* and *GPNMB* — that are artificially elevated in vitro are concurrently downregulated (Fig. 4d, e). Morphologically, these microglia develop ramified processes, establish territorial domains with appropriate nearest-neighbour distances and exhibit surveillance motility similar to endogenous cells^13,30,31^ (Fig. 4b).

Despite their similarities to primary human microglia, ICV delivery of transplanted cells limits their spatial distribution, leading them to cluster around injection sites, such as the cortex, hippocampus and striatum^13,30,31^. This mosaic coexistence provides an internal control for comparing species-specific responses to local pathology, but restricts the study of global brain disorders. To achieve greater chimerism, transplanted cells have been engineered to resist CSF1R inhibitors by introducing a specific point mutation (G795A) into the *CSF1R*, which renders hPSC-derived microglia resistant to pharmacological CSF1R blockade^59–61^ (Fig. 4f). Prolonged CSF1R inhibitor treatment post-transplantation then selectively eliminates endogenous mouse microglia while sparing the inhibitor-resistant human cells, enabling near-complete chimerism across all brain regions^59–61^.

The two engraftment approaches serve complementary purposes (Fig. 4f). Standard localized engraftment remains optimal for mechanistic studies of regional neuroimmune interactions and species-specific comparative analyses. Conversely, brain-wide replacement provides a system for evaluating microglial replacement therapies in diffuse primary microgliopathies, including adult-onset leukoencephalopathy with axonal spheroids and pigmented glia (ALSP)^60^. Achieving global distribution also enables engineered human microglia to serve as brain-wide delivery vehicles for secreted therapeutic proteins to combat widespread neurodegenerative conditions^61^.

Transplanted human microglia exhibit context-dependent responses across pathological conditions. In the *APP*^*NL-G-F*^ mouse model of AD, xenografted human microglia adopt five transcriptional programmes — homeostatic microglia, DAM, HLA, cytokine response modifier and interferon-responsive microglia^14^. In a model of frontotemporal dementia caused by progranulin deficiency, the introduction of wild type human microglia restores progranulin levels, normalizes microglial states, reduces lipofuscinosis and alleviates neuroinflammation. This, in turn, rescues thalamic circuit hyperexcitability and ameliorates social behaviour deficits, demonstrating that microglial progranulin sustains CNS homeostasis^62^. In chimeric models of *C9orf72*-associated amyotrophic lateral sclerosis, hexanucleotide repeat expansions cause microglial haploinsufficiency. This impairs the transition to disease-associated states and induces endolysosomal dysfunction with defective cargo degradation, thereby distinguishing *C9orf72* amyotrophic lateral sclerosis from sporadic forms^63^.

Human microglia chimeric models also provide critical insights into neural development and neurodevelopmental disorders. Xenotransplantation studies reveal that human microglia retain species-specific developmental programming and region-specific morphological diversity in vivo. The human-specific expression of *SRGAP2B/C* modulates the homeostatic morphological maturation of human microglia^64^. Chimeric platforms incorporating human neurons effectively model multifactorial conditions, including ASD and Down syndrome (DS)^65,66^. Engrafting human microglia alongside human neurons demonstrates that ASD neuronal defects drive abnormal microglial states in a cell-non-autonomous manner, even without intrinsic microglial mutations. In chimeric models of DS, patient-derived microglia exhibit aberrant synaptic pruning, which disrupts neurotransmission and impairs synaptic plasticity^65,66^. Cumulatively, these models reveal the crucial role of neuroimmune crosstalk in early human brain development and disease.

In addition, chimeric models are valuable for preclinical evaluation of human-targeted therapeutics. For example, the effectiveness of lecanemab^67^ (an FDA-approved monoclonal antibody against amyloid β) was tested in *APP*^*NL-G-F*^ × *Csf1r*^ΔFIRE/ΔFIRE^ mice with hPSC-derived microglia grafts. Systemic administration significantly reduced amyloid plaques via Fc-mediated activation of human microglia; deleting the Fc fragment abolished the therapeutic effect^58^. This demonstrates the necessity of humanized chimeras for elucidating the mechanisms of therapeutic antibodies that lack cross-reactivity with murine targets.

Overall, human microglial chimeric models offer distinct advantages: preserving disease-associated human genetic backgrounds, driving in vivo maturation to transcriptomic profiles comparable with those of primary microglia, and enabling longitudinal evaluation of human-specific therapeutics. However, the rodent brain microenvironment may impose species-specific constraints on human microglia behaviour. Achieving uniform chimerism is technically challenging and requires CSF1R inhibitor-resistant strategies. Additionally, the need to use immunodeficient hosts precludes the analysis of adaptive immune contributions to neurodegeneration. Continued improvements, such as CSF1R inhibitor-resistant microglia^59–61^, will enhance translational fidelity of the chimeric microglia model and accelerate the development of targeted therapeutics for microglia-associated neurological diseases in vivo.

## Conclusion

The establishment of hPSC-derived microglial platforms facilitates the investigation of human-specific neuroimmune mechanisms governing CNS homeostasis and disease. This review traces the evolution of microglial models from in vitro cultures to in vivo chimeric systems, each offering an experimental foundation for examining human genetic risk variants within physiologically relevant settings.

Single-cell transcriptomic analyses of chimeric models demonstrate that human microglia exhibit distinct transcriptional profiles, including HLA antigen-presenting and cytokine-responsive states that are often absent or diminished in rodent models^14^. This evolutionary divergence in immune responses highlights the need for human models to properly represent the polygenic nature of late-onset neurodegenerative diseases. Advances in these models show that the microenvironment controls microglial identity. Given that isolated human microglia undergo transcriptomic drift and lose their core homeostatic signatures^8,12,26–28^, integrating these cells into complex environments confirms that cellular context dictates their functional state. Three-dimensional organoids provide essential neuroglial interactions and self-organized cytoarchitecture, thereby preserving homeostatic gene expression more effectively than planar culture^50–53,68^. Within xenotransplantation models, the intact brain matrix provides the physiological cues necessary to restore and sustain mature in vivo microglial phenotypes^13,14,29,66^.

Technological progress has transformed these platforms from basic observational tools into methods for the functional validation of human microglial biology. Monolayer cultures offer the experimental tractability necessary for genome-wide CRISPR screening, facilitating identification of human-specific disease modifiers, including polygenic AD risk loci that lack accurate murine orthologues^41,42^. Three-dimensional organoid systems restore neuroglial crosstalk to reverse culture-induced transcriptomic drift, providing the spatial context required to investigate human microglial synaptic pruning and early pathology within a native-like cytoarchitecture. Humanized chimeric models serve as a translational bridge, embedding human microglia into intact biological systems featuring systemic circulatory inputs and progressive ageing. Integrating these platforms establishes a continuous pipeline for transitioning from high-throughput discovery of human microglial targets to in vivo preclinical validation.

Despite rapid advancements, critical challenges remain in accurately modelling human microglia. Current hPSC-derived protocols yield microglia that retain fetal or early postnatal transcriptomic signatures, necessitating the incorporation of epigenetic ageing or cellular senescence to capture the dystrophic and lipid-accumulating microglial phenotypes that drive late-onset neurodegeneration. Given that local neuronal cues dictate microglial spatial heterogeneity, multicellular platforms dominated by cortical lineages fail to model region-specific microglial vulnerabilities across distinct anatomical structures^69^. Blood–brain-barrier integrity, circulating metabolites and peripheral immune cells profoundly shape microglial activation states in the intact brain. Isolated in vitro models lacking vascular networks and systemic immune interactions, therefore, restrict the accurate modelling of microglial responses to peripheral inflammation. Engineering region-specific organoids that integrate vascular components and performing targeted xenotransplantation into distinct brain regions remains essential to fully recapitulate the environmental cues required for authentic microglial function.

Overall, these advanced microglial platforms establish an integrated framework bridging molecular genetics, cellular neuroscience and translational medicine. Interrogating human-specific microglial variants in physiological contexts facilitates evaluation of the polygenic architecture of microglia-driven neurodegenerative diseases, including those driven by *TREM2*. The field is now advancing beyond proof of concept to tackle previously elusive mechanistic questions, including how microglia-centred immunotherapies depend on species-specific Fc receptor interactions for target clearance. Insights gained from humanized microglia models can guide future clinical trial strategies and biomarker discovery for neuroinflammation, ultimately supporting the development of precision therapies for microglia-related neurological conditions.
